# Effectiveness of photosensitized curcumin fibers, aloevera, amla juice and panchatulsi in disinfecting guttapercha cones

**DOI:** 10.6026/973206300200620

**Published:** 2024-06-30

**Authors:** Anurag Jain, Anchita Lavania, Nayna Sharma, Saloni Goenka, Saurabh Mankeliya, Sonal Bansal, Ramanpal Singh Makkad

**Affiliations:** 1 Department of Conservative Dentistry and Endodontics, Maharana Pratap College of Dentistry and Research Centre, Gwalior, M.P., India; 2 Department of Conservative Dentistry and Endodontics, Posted in District Hospital Morar, Gwalior, M.P., India; 3 Department of Conservative Dentistry and Endodontics, Bhabha College of Dental Sciences and Research Centre, Bhopal, M.P, India; 4 Department of Oral Medicine and Radiology, New Horizon Dental College and Research Institute, Bilaspur, Chhattisgarh, India

**Keywords:** Curcumin fibres, Amla, Aloe vera, Panchtulsi, disinfection, Gutta-percha

## Abstract

Herbal remedies have demonstrated remarkable effects as anti-diabetic, anticancer, antimicrobials, immunological modulatory agent in liver problems, respiratory illnesses, and as beauty agents. The need for more affordable, readily accessible, and alternative medicines has led to a rise in the recognition of herbal drugs. Therefore, it is of interest to evaluate and compare the effectiveness of photosensitized curcumin fibers, Aloevera, Amla Juice and Pancha Tulsi in disinfecting guttapercha (GP) cones. It was observed that all experimental disinfectants were found to have greater antimicrobial action than the positive control in which no disinfectant was used. The order of antimicrobial action among different experimental disinfectants against Staphylococcus aureus and Enterococcus faecalis in disinfection of GP cones was in following order PanchaTulsi>Curcumin fibers >Amla juice > Aloe vera. It was concluded that all herbal disinfectants were found to have antimicrobial effectiveness in disinfection of GP cones with Panchtulsi having maximum disinfectant ability followed by photosensitized curcumin fibres.

## Background:

Maintaining an aseptic continuum from the point of access tooth preparation to the eventual coronal rehabilitation of the tooth is essential to ensuring the effectiveness of endodontic therapy [1, 2- 3]. In addition to natural oral microbial fauna, the practitioner has to be aware of external contamination by bacteria. Every tool and substance inserted into the root canals ought to remain sterile for the best possible protection against infection [4, 5-6]. This also applies to obturating elements. The most utilized core material for root canal system obturation is GuttaPercha (GP) cones [7, 8-9]. Despite being produced in an aseptic environment and having possible antibacterial qualities due to the zinc oxide ingredient, aerosols, incorrect preservation, and improper handling may contamination GP cones [10, 11-12].Studies have shown that the most frequent microorganism infecting GP cones within the boxes and during glove interaction is the Staphylococcus genus [13, 15-16]. According to a study, there is a 15.7 percent genus of Staphylococcus genus, which supports the need for general purification [17, 18- 19]. According to a number of investigations, E. faecalis is the most resilient intracanal pathogenic organism in failures of root canal therapy and is considered to be the gold-standard bacteria in endodontic studies [20, 21, 22- 23]. Because of its greater infectiousness, E. faecalis was chosen for the purpose of investigation to serve as one of the other potential microorganisms that could infect GP cones [24, 25]. Since endodontic therapy primarily involves decontamination to stop the spread of microorganisms all through the system of root canals and tissues at periapical area, it would be beneficial if the obturation material employed for filling the root canal framework was free of bacteria that are pathogenic [12- 16]. Therefore, it is crucial to quickly sterilize GP cones on the chair side in order to preserve the aseptic sequence throughout root canal therapy [11-17]. Because GP cones are heat labile, it is not possible to apply moist or dry heat sterilization on them because it will change their GP structure [19-23]. As a result, chemically-based disinfectants such sodium hypochlorite, paraformaldehyde, ethyl alcohol and formocresol are frequently employed in cold the sterilization process, which typically takes one to five minutes to disinfect [13- 18]. Sodium hypochlorite is the most efficient of all because it disinfects in just one minute. However, the deposition of crystals on the surface of GP cone is recorded at every concentrations, which weakens the sealers' binding with the GP cones and causes microleakage[19-24].Curcumin, a chemotherapeutic agent derived from root of turmeric plant, constitutes one of the naturally occurring substances with known antiinflammatory and anti-microbial qualities [2-4]. It also has anticancer and antimicrobial activities, making it an important therapeutic possibility for both the avoidance and management of various illnesses [5-9]. In recent times, curcumin has been successfully utilized in the form of intracanal endodontic irrigant during endodontic therapy, demonstrating beneficial and successful disinfection outcomes, which are likely due to its permeabilization implications, which damage the membranes of bacteria [3-6]. Additionally, curcumin is has photosensitive properties and, in a research, the combination of curcumin irrigation for infection-ridden root canals as well as photoactivation (5 min) utilizing an LED unit significantly decreased infection [5-9]. Commonly found in the Indian market, herbal remedies have demonstrated remarkable effects as anti-diabetic, anticancer, antimicrobials, immunological modulatory agent in liver problems, respiratory illnesses, and as beauty agents [10- 18]. The need for more affordable, readily accessible, and alternative medicines has led to a rise in the recognition of herbal drugs [12-19].Therefore, it is of interest to evaluate and compare the effectiveness of curcumin fibers with photoactivation, Aloevera Juice, Amla Juice and Pancha tulsi in disinfecting GP cones.

## Methods and Materials:

It was an *in vitro* study. The investigation comprised 220 GP cones (Dentsply, Size 80, and 2% taper) that were removed from recently opened boxes while still under sterile conditions. Broken and warped cones were thrown away. The test organisms that were employed were a microbial sample of E. faecalis, whose strain specification was ATCC2912, and S. aureus, whose strain specification was ATCC6538, with an estimated concentration of 108 CFU/ml in Trypticase Soy Broth. The herbal solutions used for the study were curcumin fibres activated by photosensitization, amla solution, aloe vera and Panchtulsi.

## Artificial infection of GP cones:

Two sets of 100 GP cones each, Category P and Category Q, were created from the 200 GP cones. For thirty minutes, Group P, which contained one hundred cones, was exposed to 20 milliliters of S. aureus microbiological suspension. For 30 minutes, Group Q, which consisted of 100 cones, was exposed to 20 milliliters of E. faecalis microbial suspension.

## GP cones were disinfected:

Following a fake infection, GP cones were submerged for a minute in each of the three disinfection treatments (amla juice, aloe vera, and panchtulsi). When it came to curcumin fibers, GP cones containing curcumin fibers were photo activated for five minutes applying LED curing equipment, which has a wavelength ranging from 385-515 nm. The GP cones from the two categories were separated into five subcategories, each containing 20 cones, according on the disinfectant that was applied [Table 1].

Each cone was placed into a sterile test tube with ten milliliters of thioglycolate medium inside it. The test tubes were then incubated for a period of one week at 37 degrees Celsius. The thioglycolate medium was transferred to a petri dish harboring Brain Heart Infusion (BHI) agar using a micropipette after seven consecutive days of incubation. The thioglycollate medium was applied thinly to the BHI agar using a sterile cotton tip. Following a 48-hour aerobic incubation period at 37 degrees Celsius, the plates were inspected using a digital colony counter to determine the number of Colony Forming Units (CFU).

## Statistical Analysis:

Version 21.0 of the Statistical Package for Social Sciences (SPSS) computer program was used to statistically evaluate the results collected. The data were analyzed using One Way Analysis of Variance (ANOVA) and the Tukey Posthoc test. A significant threshold of p <0.01 was applied.

## Results:

The mean CFU (x10-1) of Enterococcus faecalis after treating GP with curcumin, Aloe vera, Amla juice, PanchaTulsi, positive control and negative control was 29.01 ±2.57, 104.10±1.85, 67.21±1.57, 2.31±0.06, 153.21 ±11.14 and 1.52± 1.11 respectively (Table 2). PanchaTulsi showed maximum antimicrobial action against Staphylococcus aureus and Enterococcus faecalis followed by curcumin fibers. Minimum antimicrobial action was exhibited by Aloe vera followed by Amla juice. It was observed that all experimental disinfectants were found to have greater antimicrobial action than the positive control in which no disinfectant was used. The order of antimicrobial action among different experimental disinfectants against Staphylococcus aureus and enterococcus faecalis in disinfection of GP cones was in following order Panchatulsi>Curcumin fibers <Amla juice > Aloe vera (Table 3).

## Discussion:

The success of endodontic therapy depends on maintaining an aseptic continuity from the moment of access tooth preparation to the tooth's final coronal restoration. The dentist must be mindful of exogenous bacterial contamination in addition to the normal oral microbial fauna [14-16]. For optimal protection against infection, every instrument and material placed into the root canals should be kept sterile [16- 19]. This is true for components that obturate as well. GuttaPercha (GP) cones are the most commonly used core material for root canal system obturation [14-21]. Even though zinc oxide is used in the production process to create aseptic conditions and may have antibacterial properties, aerosols, poor handling, and inappropriate preservation can contaminate GP cones. Herbal treatments, which are widely available in our market, have shown amazing results as anti-inflammatory, anti-cancer, anti-diabetic, and immunomodulatory agents in respiratory and liver disorders as well as cosmetic applications [8-19]. Herbal remedies are becoming more well-known as a result of the demand for more accessible, reasonably priced, and alternative medications [12-16]. However, there is currently a paucity of knowledge regarding their use as disinfectants in endodontic therapy. Consequently, the present investigation utilized herbal medicines such as Panchatulsi, Amla Juice, Aloevera Juice, and Turmeric.

Therefore, the present study was performed to evaluate and compare the effectiveness of curcumin fibers with photoactivation, aloevera juice, amla juice and Panchatulsi in disinfecting GP cones. It was observed that PanchaTulsi showed maximum antimicrobial action against Staphylococcus aureus and Enterococcus faecalis followed by curcumin fibers. Minimum antimicrobial action was exhibited by Aloe vera followed by Amla juice. It was observed that all experimental disinfectants were found to have greater antimicrobial action than the positive control in which no disinfectant was used. Our study showed that all herbal disinfectants were found to have antimicrobial effectiveness in disinfection of GP cones with Panchtulsi having maximum disinfectant ability. These findings were also supported by findings of other studies. According to the results of this investigation, PanchaTulsi exhibited superior disinfectant activity in comparison to Amla and Aloevera juices. Compared to amla juice and aloevera juice, panchatulsi would have had a stronger disinfecting effect due to the presence of an apparent higher quantity of bioactive components [12- 17].Given that the main goal of endodontic therapy is to decontaminate the tissues surrounding the tooth, as well as the system of root canals, it would be advantageous if the obturation material used to fill the root canal framework was devoid of pathogenic bacteria [18-24]. Therefore, in order to maintain the aseptic sequence during root canal therapy, it is imperative that GP cones on the chair side be sterilized as soon as possible. Applying wet or dry heat sterilizing to GP cones is not feasible due to their heat labile nature, which alters their GP structure [16-23]. Thus, in the cold sterilization procedure, chemically based disinfectants such formocresol, ethyl alcohol, sodium hypochlorite, and paraformaldehyde are commonly used [14-19]. Since sodium hypochlorite can disinfect an area in about one minute, it is the most effective of all. Nonetheless, crystal deposition on the GP cone surface is observed at all concentrations, weakening the sealers' adherence to the GP cones and leading to microleakage[20-25].In our study, the order of antimicrobial action among different experimental disinfectants against Staphylococcus aureus and Enterococcus faecalis in disinfection of GP cones was in following order Panchatulsi > Curcumin fibres > Amla juice > Aloe vera.

The findings of this study are similar to findings of other studies [4-13]. Aloevera gel's antibacterial activity against three microorganisms was demonstrated by a study [12- 18] by the formation of effective inhibitory zones that were nearly equal to 5.25 percent NaOCl. Three herbal gels were tested for their ability to effectively disinfect GP cones in a study. They came to the conclusion that the inhibition zones on each gel were almost equal to 5.25 percent NaOCl [12-19]. Herbal products are therefore a good alternative to chemical disinfectants. Our study found that curcumin fibres had second best antimicrobial properties regarding disinfection of GP cones. This observation was in accordance with the findings of other studies [15-24]. Curcumin, a chemotherapeutic agent derived from root of turmeric plant, constitutes one of the naturally occurring substances with known anti-inflammatory and anti-microbial qualities [3-8]. It also has anticancer and antimicrobial properties, making it an important therapeutic possibility for avoidance and management of various illnesses. In recent times, curcumin has been successfully utilized in the form of intracanal endodontic irrigating agent during endodontic therapy, demonstrating beneficial and successful disinfection outcomes, which are likely due to its permeabilization implications that damage the membranes of bacteria [6-14]. Additionally, curcumin has photosensitive properties and, in a research, the combination of curcumin irrigation for infection-ridden root canals as well as photoactivation (5 min) utilizing an LED unit significantly decreased infection [18-25].

## Conclusion:

Data shows that all herbal disinfectants were found to have antimicrobial effectiveness in disinfection of GP cones with panchtulsi having maximum disinfectant ability followed by curcumin fibres activated by photosensitization.

## Figures and Tables

**Table 1:** Distribution of study specimens

**Category**	**Subcategory**	**Bacteria for contamination**	**Disinfectant used**	**Number**
**P**	**1 (positive control)**	**Staphylococcus aureus**	**No**	**20**
	2	Staphylococcus aureus	Curcumin fibres photosensitized	20
	3	Staphylococcus aureus	Amla juice	20
	4	Staphylococcus aureus	Aloe vera	20
	5	Staphylococcus aureus	Panchtulsi	20
**Q**	**1**	**E faecalis**	**No**	**20**
	2	E faecalis	Curcumin fibres photosensitized	20
	3	E faecalis	Amla juice	20
	4	E faecalis	Aloe vera	20
	5	E faecalis	Panchtulsi	20
Negative control	Uncontaminated GP cones			20

**Table 2:** Data showing mean number of colonies (x10-1) among the different disinfecting solutions treated against Staphylococcus aureus

	**Positive control**	**Negative control**	**Curcumin**	**Aloe vera**	**Amla juice**	**Pancha Tulsi**
Mean± SD	131.81 ±145.0	1.17±0.41	17.47±1.14	99.71 ± 7.1	65.59 ±2.19	2.12±0.79
F	397.7					
P value	0.001					
The mean CFU (x10-1) of Staphylococcus aureus after treating GP with Curcumin, Aloe vera, Amla juice, PanchaTulsi, positive control and negative control was 17.47±1.14, 99.71 ± 7.1, 2.12±0.79, 131.81 ±145.0 and 1.17±0.41 respectively (Table 2).

**Table 3:** Data showing mean number of colonies (x10-1) among the different disinfecting solutions treatedagainst Enterococcus faecalis

	**Positive control**	**Negative control**	**Curcumin**	**Aloe vera**	**Amla juice**	**PanchaTulsi**
Mean± SD	153.21 ±11.14	1.52± 1.11	29.01 ±2.57	104.10±1.85	67.21±1.57	2.31±0.06
F	397.7					
P value	0.001					
